# Evaluation of novel beverage formulations for hydration enhancement in humans

**DOI:** 10.2478/joeb-2023-0002

**Published:** 2023-04-26

**Authors:** Grant M. Tinsley, Madelin R. Siedler, Christian Rodriguez, Patrick S. Harty, Matthew T. Stratton, Sarah J. White, Dale S. Keith, Jacob J. Green, Jake R. Boykin, Abegale D. Williams, Brielle DeHaven, Alexandra Brojanac, Ethan Tinoco

**Affiliations:** 1. Energy Balance & Body Composition Laboratory; Department of Kinesiology & Sport Management, Texas Tech University, Lubbock, TX, USA; 2. Department of Kinesiology; College of Science, Technology, and Health; Lindenwood University, St. Charles, MO, USA; 3. Department of Health, Kinesiology and Sport; University of South Alabama, Mobile, AL, USA

**Keywords:** Hydration, bioelectrical impedance analysis, bioimpedance, water

## Abstract

This study evaluated the influence of novel beverage formulations on bioimpedance- and urine-based hydration markers. Thirty young healthy adults (n=16 females, n=14 males; age: 23.2±3.7 years; BMI: 24.3±3.3 kg/m^2^) participated in a randomized, double-blind, placebo-controlled, crossover study. Participants completed three conditions with baseline bioimpedance, urine, and body mass assessments, followed by ingestion of one liter of a test beverage over a 30-minute period. The three beverages were: active hydration formulation in still (AFstill) or sparkling (AFspark) water and a still water control. The active formulations were identical in concentrations of alpha-cyclodextrin and complexing agents. Following beverage ingestion, bioimpedance assessments were performed every 15 minutes for two hours, followed by final urinary and body mass assessments. The primary bioimpedance outcomes were phase angle at 50 kHz, resistance of the extra-cellular compartment (R_0_), and resistance of the intracellular compartment (Ri). Data were analyzed using linear mixed effects models, Friedman tests, and Wilcoxon tests. Statistically significant changes in phase angle values were observed at 30 (p=0.004) and 45 minutes (p=0.024) following the initiation of beverage ingestion in the AFstill condition as compared to the reference model (i.e., control condition at baseline). Although differences between conditions were not statistically significant at later time points, the data were consistent with AFstill having greater elevations in phase angle throughout the monitoring period. At the 30-minute time point only, statistically significant differences in R_0_ for AFspark (p<0.001) and in Ri for AFstill (p=0.008) were observed. When averaged across post-ingestion time points, there was a trend (p=0.08) for Ri differences between conditions. The net fluid balance was greater than zero, indicating retention of ingested fluid, for AFstill (p=0.02) and control (p=0.03), with a trend for AFspark (p=0.06). In conclusion, an active formulation containing alpha-cyclodextrin in still water demonstrated potential benefits for enhancing hydration markers in humans.

## Introduction

Among medical professionals and health-conscious consumers, there is sustained interest in understanding and implementing optimal hydration strategies. Estimates from the National Health and Nutrition Examination Survey (NHANES) indicate that approximately 33 to 55% of individuals in the United States may be inadequately hydrated [1,2]. In the UK, it was estimated that 37% of patients admitted to a hospital were dehydrated [3]. While inadequate hydration can exert ill effects at any age, children and older adults may be particularly susceptible, along with those with chronic illnesses like diabetes, cystic fibrosis, or kidney problems and those taking medications influencing urination or sweat rates [4]. Causal links continue to be investigated, but associations between inadequate hydration and numerous disease states are now recognized [5,6]. For example, correlations between fluid intake and kidney health – including the development of chronic kidney disease and occurrence of kidney stones – and metabolic health, such as glycemia, have been observed [6]. In addition to general health and disease states, hydration is a critical consideration during exercise and sport, in which dehydration is known to negatively influence several aspects of physical performance [7, 8]. Hydration assessment in sport also demonstrates the challenges and limitations associated with using singular fluid balance assessments, such as short-term body mass changes or urinary measures, to establish dehydration. In this regard, methods have been proposed to integrate multiple hydration-related outcomes – such as changes in body mass, urine color, and thirst level – to provide a practical method of assessing hydration status [9, 10].

Despite a general awareness of the importance of hydration for optimal physiological function and overall health, there is not a clear scientific consensus regarding fluid intake guidelines [11]. While total fluid intake is undoubtedly a major component of hydration, the specific hydrating properties of different beverage formulations may also be influential. In a seminal investigation, Maughan et al. [12] defined the short-term hydrating properties of numerous commonly consumed drinks through development of the beverage hydration index (BHI), calculated as mass of urine excreted in the two hours following ingestion of one liter of still water divided by the mass of urine excreted in the two hours following ingestion of one liter of an alternate beverage. While traditional beverages – such as milk, cola, tea, coffee, sports drinks, and juice – were investigated, novel beverages designed to promote hydration are increasingly available to consumers and have yet to be studied in this manner. In this regard, several compounds have demonstrated potentially meaningful effects on cellular hydration markers.

**Fig. 1:**
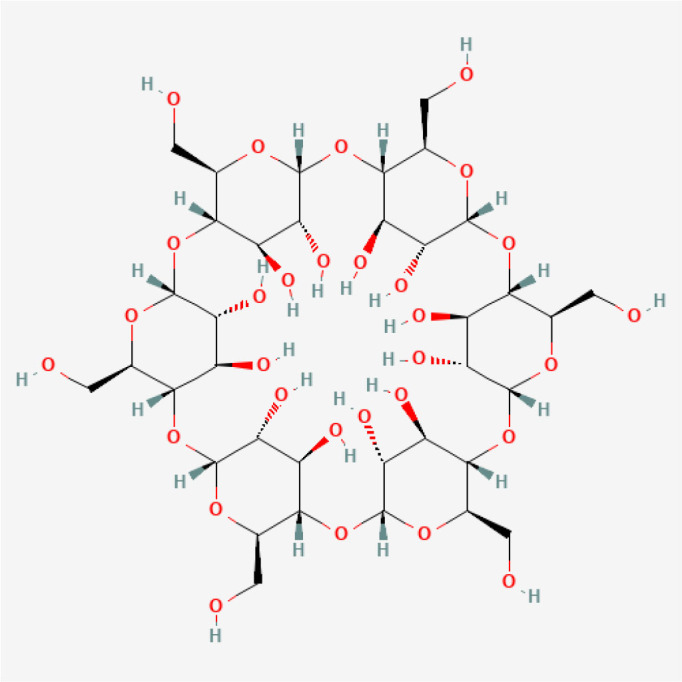
**Chemical structure of alpha-cyclodextrin**. *Image courtesy of pubchem.ncbi.nlm.nih.gov*.

One particularly interesting carbohydrate, alpha-cyclodextrin (i.e., cyclomaltohexaose; Figure 1), was recently demonstrated to enhance water uptake through human water channels, aquaporins, expressed in a single-cell *Xenopous* oocyte model [13]. Furthermore, the same investigation reported a significantly longer lifespan in *Caenorhabditis elegans*, a multicellular organism commonly used to study aging, following treatment with alpha-cyclodextrin [13]. Preliminary work in humans has also demonstrated the potential for alpha-cyclodextrin-containing beverages to increase phase angle, a bioimpedance-based marker of cellular hydration and health [14].

While the importance of hydration for human health and wellbeing is recognized, the evaluation of hydration itself is a notable challenge, with no current “gold standard” method [15]. Traditionally, investigations have relied on whole-body, hematologic, or urinary methods [12, 15]. However, other methods have been proposed and implemented. One such technique that may be particularly notable is bioimpedance. While fluid volumes – including total body water, intra-cellular water, and extracellular water – can be estimated through bioimpedance, compelling arguments for the use of raw bioimpedance metrics (i.e., phase angle, resistance, and reactance) for the evaluation of hydration in humans have been proffered [16].

Specifically, the assumptions necessitated by estimating fluid volumes call into question their sensitivity to detect individual changes. In contrast, raw bioelectrical properties can be quantified without transformation or assumptions. Of raw bioimpedance metrics used in clinical nutrition settings, phase angle is the most established. This value is thought to reflect both the quantity and quality of soft tissue in the body and is commonly investigated for its relationship to mortality and disease progression [17]. Furthermore, it has been used as a noninvasive marker of cell membrane integrity and cell function, with higher values indicating better function and health, as well as a marker of hydration [17]. However, other raw bioimpedance metrics may provide more specific information about the intracellular and extracellular fluid compartments. For example, modeled resistance at zero frequency (R_0_) represents resistance of the extracellular compartment, while modeled resistance at an infinite frequency can be used to estimate resistance of the intracellular compartment (Ri) [16].

Based on the prevalence of inadequate hydration among the general population, the ill effects of suboptimal hydration, and the strong interest in optimal hydration strategies expressed by health-conscious consumers, the investigation of novel hydration formulations is warranted. And due to debate concerning the best methods of hydration assessment, investigations implementing assessments based on a combination of multiple factors – such as bioimpedance and urinary markers – are warranted. Therefore, the aim of the present investigation was to determine if novel beverage formulations containing alpha-cyclodextrin as the primary ingredient improve bioimpedance- and urine-based hydration markers in humans. It was hypothesized that the novel formulations would improve hydration as indicated by these markers.

## Materials and methods

### Overview and Participants

This study was a randomized, double-blind, placebo-controlled crossover trial. Each participant completed three conditions, each of which involved serial bioimpedance assessments following ingestion of 1 L of water, with or without active formulation ingredients. Individuals were eligible for participation if they were between the ages of 18 and 40, generally healthy, and had a body mass index <30 kg/m^2^. Individuals were ineligible if they had a history of cardiovascular, renal, musculoskeletal, or metabolic disease; had a history of incontinence, frequent urination, or other urination-related reports that would make the study protocol difficult to complete; had a pacemaker or other electrical implant; had prior amputation of the hand or foot; or were currently pregnant. All participants read and signed a university-approved consent document prior to participation. Following consent, each participant was assigned to complete the study conditions in a randomized order.

Thirty participants, including 16 females and 14 males, completed the study. Nineteen participants were non-Hispanic Caucasians, 4 were Hispanic Caucasians, 4 were Asian, 2 were South American, and 1 was Black. Additional participant characteristics are displayed in Table 1.

**Table 1:** **Participant characteristics.** Thirty participants (n=16 females; n=14 males) completed the study.

	Mean ± SD
**Age (y)**	23.2 ± 3.7
**Height (cm)**	169.2 ± 7.9
**Weight (kg)**	70.2 ± 13.8
**BMI (kg/m^2^)**	24.3 ± 3.3
**Body fat (%)**	24.1 ± 8.5

### Informed consent

Informed consent was obtained from all individuals included in this study.

### Ethical approval

The research related to human use complied with all relevant national regulations, institutional policies and in accordance with the tenets of the Helsinki Declaration, and has been approved by the authors’ institutional review board or equivalent committee (protocol ID: IRB2020-527).

### Laboratory Procedures

Upon reporting to the laboratory, participants were interviewed to confirm adherence to the pre-assessment restrictions, which included abstention from eating, drinking, or consuming nicotine or medication for at least 8 hours, and abstention from alcohol consumption and exercise or vigorous physical activity for at least 24 hours. At the first study visit, participants also completed a dietary record, which was provided to them to facilitate replication of the same dietary intake for the one day preceding the subsequent two study visits.

Each participant was then asked to void their bladder and provide a urine sample for subsequent assessment of urine specific gravity (USG) via digital refractometer (PA201X-093, Misco, Solon, OH, USA). Thereafter, a baseline body mass assessment was performed via calibrated scale (BWB-627-A, COSMED USA, Inc., Concord, CA, USA), along with assessment of height via mechanical stadiometer. Following these assessments, participants completed 10 minutes of supine rest before a baseline assessment via bioimpedance spectroscopy (BIS; ImpediMed SFB7, Carlsbad, CA, USA).

The BIS analyzer utilizes 256 measurement frequencies ranging from 3 to 1000 kHz and a unilateral hand-to-foot electrode arrangement. This device was checked using the manufacturer-provided test cell prior to use. The sites for adhesive electrodes were cleaned with alcohol wipes prior to placement of the electrodes. The proximal wrist electrode was placed between the styloid processes of the radius and ulna bones, and the distal wrist electrode was placed 5 cm distal to the proximal electrode. For the ankle, the proximal electrode was placed between the medial and lateral malleoli of the tibia and fibula bones, and the distal ankle electrode was placed 5 cm distal to the proximal electrode. Additionally, the legs were positioned to ensure they did not touch, and the arms were separated from the torso by an ^~^30° angle. BIS output was reviewed for quality assurance through visual inspection of Cole plots. For the present analysis, phase angle values were specified a priori as the bioimpedance outcome of interest. In an independent sample of 18 participants, our laboratory’s technical error of measurement for BIS phase angle was 0.01° (0.17%).

After the baseline BIS assessment, participants ingested 1 L of one of three test beverages over the course of 30 minutes, provided as 250-mL aliquots each 7.5-minute period. The three test beverages were the active hydration formulation in still water (AFstill), the active hydration formulation in sparkling water (AFspark), and a still water control (CON). Both active formulations contained a proprietary combination of hydration-promoting ingredients provided by the sponsor (8 POiNT, LLC). Ingredients included alpha-cyclodextrin (Cavamax W6, Wacker Chemie AG, Munich, Germany) and complexing agents (amino acids and B-vitamins; Kyowa, Tokyo, Japan). The same bottled water source was used for all beverages, and no electrolytes or other charged ionic species were added to any of the beverages. Immediately following the 30-minute ingestion period, a second BIS assessment was performed. Additional assessments were performed every 15 minutes for two hours, with the participants remaining supine for all assessments. However, in the event a participant needed to urinate before all BIS measurements had been completed, the participant was allowed to leave the supine position and void their bladder, collecting all urine. The participant then returned to the supine position for the remainder of the monitoring period, then collected any additional urine at the time the monitoring period was complete (i.e., 150 minutes after the beginning of the fluid ingestion period). However, no additional BIS assessments were performed after a participant had risen from the supine position, due to the documented effects of postural changes on bioimpedance estimates [18]. In these events, the BIS data from the last collected assessment was used for the remaining time points (i.e., last observation carried forward).

BIS data were processed using the manufacturer-provided software (BioImp, ImpediMed). Phase angle from the 50 kHz frequency was utilized, along with R_0_ – representative of resistance of the extracellular compartment – and Ri, representative of resistance of the intracellular compartment. After all BIS assessments had been completed, the participant voided their bladder and collected all urine. The volume of urine was recorded, and the USG was tested. Finally, a second body mass assessment using the calibrated scale was performed.

Following completion of the first study condition, participants returned on separate days to complete the second and third conditions. Scheduling of visits occurred without consideration of the menstrual cycle due to the short duration between visits, utilization of baseline assessments in each condition, and prior research indicating minimal influence of the menstrual cycle on bioimpedance parameters [19–21].

### Data Analysis

The BHI for AFstill and AFspark was calculated as mass of urine excreted in the two hours following ingestion of one liter of still water (i.e., control condition) divided by the mass of urine excreted in the two hours following ingestion of the active formulation beverage. Net fluid balance was calculated as the volume of ingested water (1 L) minus the total volume of urine produced during the monitoring period.

Bioelectrical data were analyzed via linear mixed effects models using the *nlme* R package. Due to repeated measures within participants, a random intercept for participant was included in the model. The model was defined as: *Phase angle*~*Condition* + *Time* + *Condition* * *Time* + ~1|*Participant*. In this model, the reference groups were control for Condition and the baseline assessment (time 0) for Time. A first-order autoregressive (AR1) variance-covariance matrix was used, along with a correlation form of *Time*|*Participant*/*Condition*. Models were fit by maximizing the restricted log-likelihood (REML). Model coefficients (i.e., *b* and associated 95% confidence intervals [CI]) were established using the *sjPlot* R package.

Outcomes with a single data point were analyzed using a non-parametric rank-based test (the Friedman test) for comparisons between groups. When necessary, post hoc tests were performed using Wilcoxon signed-rank tests. One-sample Wilcoxon tests were used for comparisons to absolute values, such as comparing BHI to a value of 1 and net fluid balance to a value of 0. Kendall’s *W* effect sizes were calculated for Friedman tests, and Wilcoxon *r* effect sizes were calculated for Wilcoxon tests. Statistical significance was accepted at *p* < 0.05, with p-values between 0.05 and 0.10 considered trends.

## Results

### Bioelectrical Variables

**Fig. 2:**
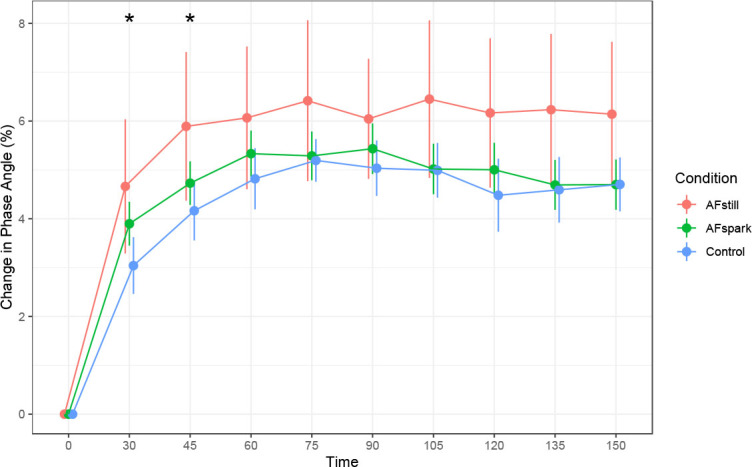
**Change in Phase Angle**. * indicates *p* < 0.05 (actual p-values were 0.004 for the 30-minute time point and 0.024 for the 45-minute time point).

Phase angle was higher at 30 and 45 minutes in the AFstill condition as compared to the reference model (Figure 2). These differences were statistically significant, and the magnitude of these effects was 1.6 to 1.7%. The differences at other time points were not statistically significant. A significant effect of time was observed at all post-baseline time points. The magnitude of increase in phase angle collapsed across conditions was 3.0 to 5.2% relative to the reference model (*see Appendix*), which was based on the baseline time point. When averaged across post-ingestion time points, there was no significant difference between conditions based on the Friedman test (p=0.90; Kendall’s *W*=0.003).

**Fig. 3:**
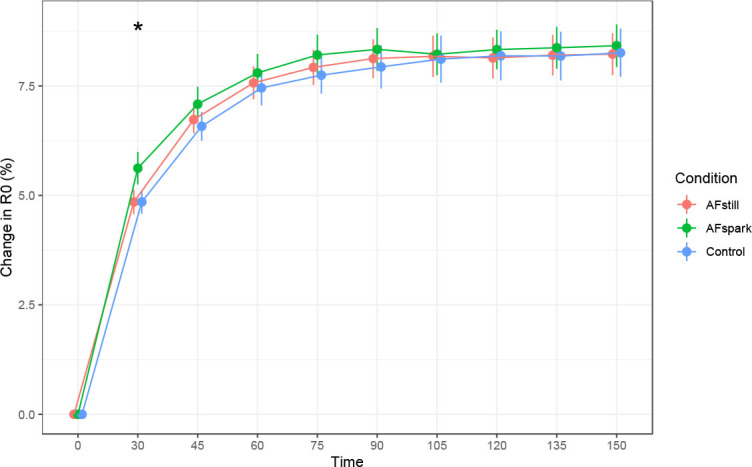
**Change in R_0_.** * indicates *p* < 0.05 (the precise p-value was *p* < 0.001).

R_0_ was higher at 30 minutes in the AFspark condition as compared to the reference model (Figure 3), without statistically significant differences between conditions at other time points. A significant effect of time was observed at all post-baseline time points. The magnitude of increase in R0 collapsed across conditions was 4.9 to 8.3% relative to the reference model (*see Appendix*), which was based on the baseline time point. When averaged across post-ingestion time points, there was no significant difference between conditions based on the Friedman test (p=0.91; Kendall’s *W*=0.003).

**Fig. 4:**
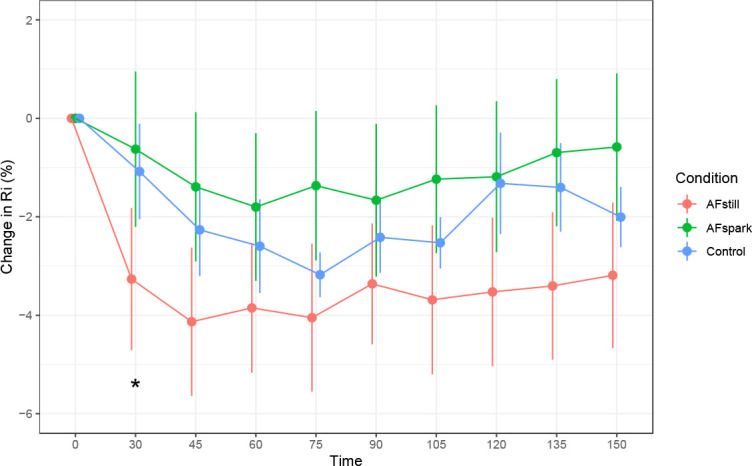
**Change in Ri.** * indicates *p* < 0.05 (the precise p-value was *p* = 0.008).

Ri was higher at 30 minutes in the AFstill condition as compared to the reference model (Figure 4), without statistically significant differences between conditions at other time points. A significant effect of time was observed for the 45, 60, 75, 90, and 105-minute time points. The magnitude of decrease in Ri collapsed across conditions was −1.1 to −3.2% relative to the reference model (*see Appendix*), which was based on the baseline time point.

When averaged across post-ingestion time points, there was a trend (p=0.08; Kendall’s *W*=0.084) for differences between conditions based on the Friedman test.

### Body Mass

**Fig. 5:**
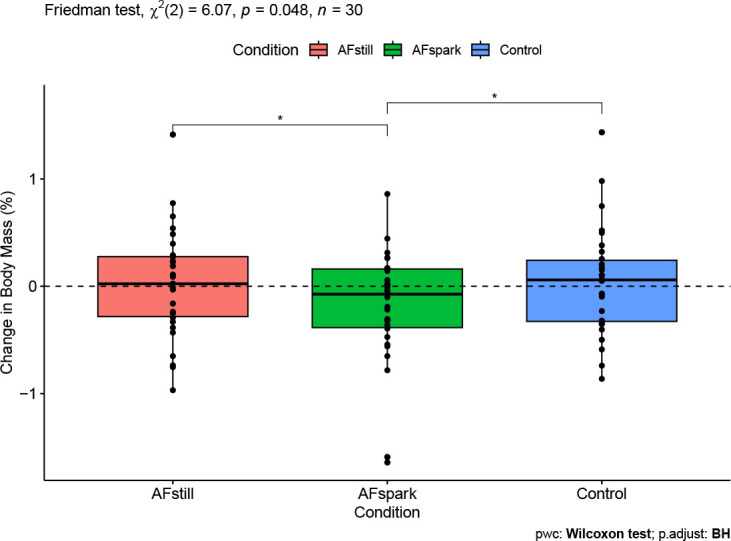
**Changes in Body Mass.** * indicates *p* < 0.05

Based on the Friedman test, percent changes in body mass differed between conditions (p=0.048; Kendall’s *W*=0.10; Figure 5). Follow-up Wilcoxon signed-rank tests indicated that body mass decreased to a greater extent in the AFspark condition ([mean ± SD] −0.19 ± 0.53%) as compared to control condition (0.04 ± 0.49%, p=0.034) and the AFstill condition (0.02 ± 0.50%, p=0.037). Wilcoxon *r* effect sizes were 0.46 for AFspark vs. AFstill, 0.41 for AFspark vs. control, and 0.18 for AFstill vs. control.

### Beverage Hydration Index

**Fig. 6:**
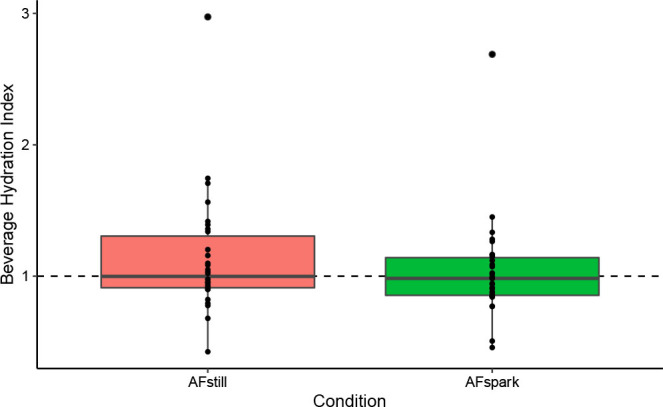
Beverage Hydration Index.

Using a one-sample Wilcoxon test, BHI did not significantly differ from 1 for AFstill ([mean ± SD] 1.13 ± 0.45; p=0.19; Wilcoxon *r* =0.17; Figure 6) nor AFspark (1.04 ± 0.38; p=0.56; Wilcoxon *r* =0.02).

### Net Fluid Balance

**Fig. 7:**
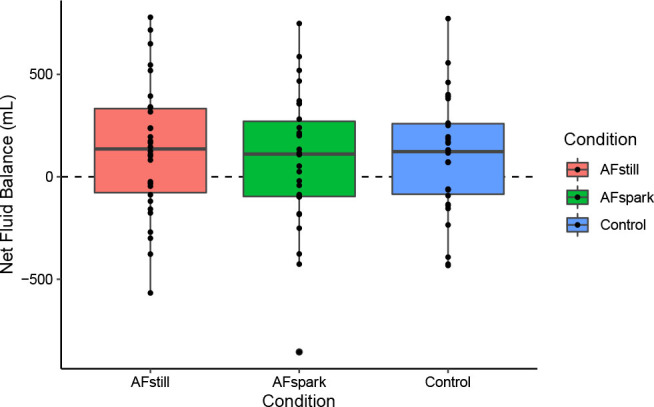
Net Fluid Balance.

One-sample Wilcoxon tests indicated that net fluid balance was greater than 0 for AFstill ([mean ± SD] 133.7 ± 323.7 mL; Wilcoxon *r*=0.38; p=0.02; Figure 7) and control (99.3 ± 288.8 mL; Wilcoxon *r*=0.34; p=0.03), with a trend for a positive net fluid balance in AFspark (79.5 ± 328.9 mL; Wilcoxon *r*=0.29; p=0.06). However, no difference between condition was observed via the Friedman test (p=0.88; Kendall’s W=0.004).

### Urine Specific Gravity

Baseline USG values were (mean ± SD) 1.0222 ± 0.0048, 1.0207 ± 0.0060, and 1.0215 ± 0.0063 in AFspark, AFstill, and control, respectively, with final USG values of 1.0035 ± 0.0013, 1.0040 ± 0.0022, and 1.0036 ± 0.0017. Via the Friedman test, there was a trend (p=0.08; Kendall’s *W*=0.10) for differences between conditions for changes in urine specific gravity (USG), with median ± IQR changes of –0.0184 ± 0.004 for AFspark, –0.0173 ± 0.0091 for control, and –0.0164 ± 0.0084 for AFstill.

**Fig. 8:**
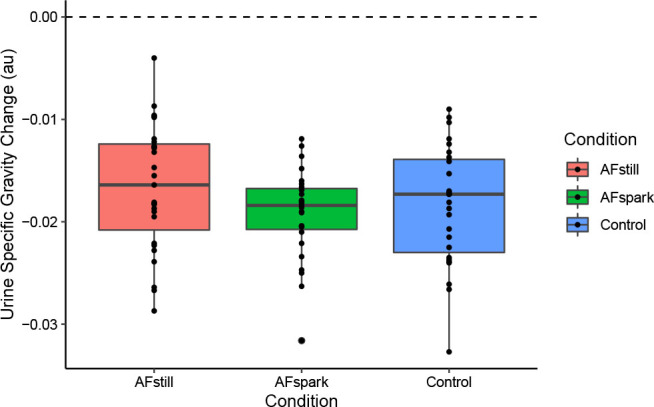
Urine Specific Gravity.

## Discussion

The present study investigated the potential hydrating effects of novel beverage formulations using multiple methods of hydration assessment. The primary bioelectrical results were that ingestion of an active formulation containing the carbohydrate alpha-cyclodextrin within still water led to a statistically significant increase in phase angle at select time points following ingestion and demonstrated a reduction in Ri, potentially indicating larger increases in intracellular fluid content relative to other conditions. Additionally, the AFstill condition exhibited a net fluid balance greater than zero, indicating retention of some of the ingested fluid two hours following intake. Some other hydration metrics – including body mass changes, urine specific gravity, and beverage hydration index – did not indicate such benefits. However, due to the lack of consensus regarding a single “gold standard” assessment [15], this investigation implemented multiple forms of hydration assessment rather than only conventional urine or blood biomarkers, as discussed below.

The prevalence of inadequate hydration and implications of optimal hydration for human health demonstrate the need for additional hydration research [1,2,5,6]. In addition to a continued examination of longstanding questions, such as simple fluid intake recommendations [11], there is sustained interest in novel compounds that may exert unique hydrating effects. The primary ingredient of the active formulations investigated in the present study was the carbohydrate alpha-cyclodextrin, which is a commonly used additive for stabilizing, emulsification, and other purposes and is generally recognized as safe (GRAS) by the US Food & Drug Administration [22,23]. The interest in cyclodextrins for hydration enhancement is based on the biophysical properties of these molecules, particularly their potential to increase water permeability and protect against cellular dehydration [13,24,25]. Alpha-cyclodextrin exhibits greater solubility in water than beta-cyclodextrin and may influence the local water structure through self-assembly into aggregated particles due to intermolecular hydrogen bonds [26,27]. Furthermore, it has been hypothesized that alpha-cyclodextrins uniquely interact with phospholipids in the cell membrane in a way that favors opening and optimal functioning of aquaporins, the specialized channels that transport water across the cell membrane in a rapid and regulated manner [13,28]. Based on this line of reasoning, the potential hydrating properties of alpha-cyclodextrin formulations were recently investigated using single-cell *Xenopus* oocytes and multicellular *C. elegans* [13]. In this investigation, significantly improved water permeability and uptake by *Xenopus* oocytes expressing human aquaporin 1 was observed following treatment with alpha-cyclodextrin formulations, as compared to water alone. This was presumably due to the effects of alpha-cyclodextrin on human aquaporin 1 activity. Additionally, lifespan of *C. elegans* was increased following treatment with the active formulations. Finally, preliminary research in humans demonstrated the potential for alpha-cyclodextrin-containing beverages to increase phase angle more than still water following acute consumption [14]. Collectively, these results provided the rationale for the present investigation in human subjects.

It has been noted that all hydration assessments provide a single measure that cannot adequately describe the complex, dynamic state of fluid compartments within the body [15]. The physiological regulation of water turnover is multifaceted and involves renal, thirst, and sweat gland responses. Furthermore, a wide variety of methodological challenges and considerations complicate the use of each individual hydration assessment, as well as comparisons between methods [15]. It should be noted that all laboratory methods of hydration assessment, with the exception of two frequently inaccessible techniques (neutron activation analysis and isotope dilution), have previously been deemed to be “based on inconsistent/limited-quality data” with “no/questionable reference criteria,” and “no/questionable validation methods” [15]. Based on these considerations, multiple metrics of hydration were employed in the present investigation in an attempt to provide as comprehensive of assessment as possible given the inherent limitations of each individual technique for hydration monitoring.

The hydration assessment methods presently employed were based on bioimpedance, body mass changes, or urinary metrics. While bioimpedance can be used to estimate fluid volumes, we elected a priori to instead examine raw bioimpedance metrics, based on strong evidence supporting the use of raw bioimpedance rather than subsequently predicted values [16]. In the present study, phase angle significantly increased to a greater extent at 30 and 45 minutes following the initiation of fluid ingestion in the AFstill condition. Although differences between conditions were not statistically significant at later time points, data were consistent with AFstill having greater elevations in phase angle throughout the monitoring period (Figure 2). The visual pattern of R0 increases during the measurement period mirrored those of phase angle. At 30 minutes following the initiation of fluid ingestion, R_0_ was higher in AFspark as compared to the reference model, indicating higher extracellular resistance. Notably, Ri was lower in AFstill at the 30-minute time point, indicating lower intracellular resistance and supporting a potential increase in intracellular fluid in this condition, with the data being consistent with this finding for the remainder of the monitoring period, despite a lack of statistical significance at later time points. While 30 to 45 minutes following initiation of beverage ingestion could be viewed as a rapid change in bioelectrical variables, these data are consistent with the swift appearance of ingested water following an overnight fast. Péronnet et al. [29] utilized water labeled with D_2_O to demonstrate that ingested water appeared in the plasma and blood cells of humans within 5 minutes of consumption, with a half-life of absorption of approximately 11 to 13 minutes. These researchers additionally demonstrated a rapid distribution of water to the central compartment, then to a peripheral compartment (half-life of 12.5 ± 4.3 min), using a two-compartment pharmacokinetic model. While the greater acute changes in phase angle and Ri can be viewed as a positive result for the active formulation, it should also be noted that this study is one of the first to apply raw bioelectrical variables for acute hydration monitoring rather than conventional urine and blood biomarkers. However, some previous investigations have used raw bioimpedance to evaluate body fluid changes in a variety of contexts, ranging from models of sepsis [30] to acute water ingestion [18,31]. Future research will likely continue to clarify the optimal methods of using raw bioimpedance for acute hydration monitoring through serial assessments.

The outcomes of body mass changes and net fluid balance were conceptually related, with both focusing on simple conservation of mass principles. Body mass changes have long been employed for acute dehydration monitoring [15], but the present investigation alternatively applied the metric to indicate fluid retention. While a significant difference in body mass changes between conditions was observed, this was driven by a larger body mass decrease in AFspark as compared to control and AFstill. Although this could be interpreted to indicate a potential diuretic effect of the sparkling water formulation, prior research by Maughan et al. [12] indicated that the BHI for sparkling water did not differ from 1 (i.e., did not differ from still water). Likewise, the present investigation found that the BHI value of AF spark (1.04 ± 0.38) and AFstill (1.13 ± 0.45) did not significantly differ from a value of 1. Furthermore, the net fluid balance – calculated as the volume of ingested water minus the volume of urine produced – indicated retention of approximately 134 mL of water, on average, two hours following completion of beverage consumption in the AFstill condition, as compared to approximately 99 mL in control and 80 mL in AFspark.

Urine specific gravity is one of the most commonly implemented urinary hydration measures, and sets of reference values are often used to evaluate the relative hydration of individuals in research and clinical settings [32]. However, the appropriateness of USG for acute hydration monitoring following beverage ingestion is questionable. Prior work has demonstrated that the consumption of a large bolus of water causes production of a high volume of dilute urine prior to equilibration of the intracellular and extracellular spaces, even in the presence of dehydration [15]. In the present investigation, fluid intake occurred over a 30-minute time period and as four separate aliquots, rather than as a single bolus. Nonetheless, comparing the dramatic decreases in USG in all conditions, which would typically be interpreted as improved hydration, with the other hydration metrics indicates the likely inappropriateness of this metric for acute hydration monitoring. For example, in the still water (control) condition, there was no change in body mass over the duration of 2 hours (percent change: 0.02 ± 0.50%), indicating that the mass of water ingested, and the mass of water excreted, primarily as urine, were essentially equal, indicating no net hydrating effect of the ingested still water over the monitoring period for this metric. In contrast, large changes in USG were observed in the control condition, with a decrease from a baseline value of 1.022 ± 0.006 to a final value of 1.004 ± 0.002. This represents a change from the category of “well hydrated” to “extremely hyperhydrated” by one set of reference values [32]. As such, the dissonance between USG and other metrics lends further support to the lack of utility of this outcome following acute fluid ingestion.

Several limitations of the present work should be acknowledged. First, as discussed, there are inherent limitations to hydration assessment due to the complex, dynamic nature of body fluids. While select techniques, such as dilution methods, can be produce accurate values when employed in select research settings, these cannot be implemented within the context of serial assessments, as in the present study. Second, the monitoring time period used in the present study was largely driven by the duration needed for calculation of BHI [12]. Longer monitoring periods may influence the magnitude of observed changes. Third, the present acute trial does not provide information regarding long-term health consequences of the novel beverage formulations. As such, future work could include chronic consumption alongside monitoring of relevant health outcomes that could be influenced by hydration. Fourth, detailed information about medication and substance use was not collected. While the generally healthy participants and acute period of abstention from medications and other substances may have minimized potential effects, some medications are known to exert effects on body fluids. Finally, the sample in the present study was composed of young, healthy individuals. Work in specific clinical populations would be needed to establish any putative therapeutic effects of the beverage formulations in these populations.

## Conclusion

A still water beverage containing alpha-cyclodextrin complexed with unique vitamins and amino acids as the primary ingredients exhibited the potential to enhance hydration. These results build upon recent work in *Xenopus* oocytes, *C. elegans*, and humans and further demonstrate the hydration benefits that cyclodextrin-containing beverages may provide as well as the need for continued research in the development and implementation of multiple methods to assess hydration biomarkers in humans.

## Appendix

**Table:** Mixed Model Coefficients for Changes in Phase Angle, R_0_, and R_i_. The table below displays mixed model coefficients, with values presented in % change relative to baseline.

	Phase Angle			R_0_			Ri		
*Predictors*	*Estimates*	*CI*	*p*	*Estimates*	*CI*	*p*	*Estimates*	*CI*	*p*
(Intercept)	0	−1.62 – 1.62	1	0	-0.76 – 0.76	1	0	−2.19 – 2.19	1
Condition [AFstill]	0	−2.05 – 2.05	1	0	−0.96 – 0.96	1	0	−2.99 – 2.99	1
Condition [AFspark]	0	−2.05 – 2.05	1	0	−0.96 – 0.96	1	0	−2.99 – 2.99	1
Time [30]	3.04	2.26 – 3.82	**<0.001**	4.85	4.55 – 5.15	**<0.001**	−1.08	−2.22 – 0.06	0.062
Time [45]	4.16	3.10 – 5.22	**<0.001**	6.58	6.17 – 6.99	**<0.001**	−2.27	−3.81 – −0.72	**0.004**
Time [60]	4.82	3.57 – 6.07	**<0.001**	7.45	6.96 – 7.95	**<0.001**	−2.6	−4.42 – −0.77	**0.005**
Time [75]	5.19	3.80 – 6.59	**<0.001**	7.74	7.19 – 8.30	**<0.001**	−3.18	−5.21 – -1.14	**0.002**
Time [90]	5.04	3.53 – 6.54	**<0.001**	7.94	7.33 – 8.54	**<0.001**	−2.42	−4.62 – -0.22	**0.031**
Time [105]	4.99	3.39 – 6.59	**<0.001**	8.11	7.46 – 8.76	**<0.001**	−2.53	−4.86 – -0.20	**0.033**
Time [120]	4.48	2.81 – 6.15	**<0.001**	8.19	7.50 – 8.87	**<0.001**	−1.32	−3.76 – 1.12	0.287
Time [135]	4.59	2.86 – 6.32	**<0.001**	8.18	7.47 – 8.90	**<0.001**	−1.4	−3.93 – 1.12	0.275
Time [150]	4.7	2.92 – 6.48	**<0.001**	8.26	7.51 – 9.00	**<0.001**	−2.01	−4.60 – 0.59	0.13
Condition [AFstill] * Time [30]	1.62	0.52 – 2.72	**0.004**	0	−0.42 – 0.42	0.993	−2.18	−3.79 – −0.58	**0.008**
Condition [AFspark] * Time [30]	0.86	−0.24 – 1.96	0.127	0.77	0.35 – 1.19	**<0.001**	0.45	−1.15 – 2.06	0.579
Condition [AFstill] * Time [45]	1.73	0.23 – 3.23	**0.024**	0.15	−0.43 – 0.73	0.607	−1.87	−4.05 – 0.32	0.095
Condition [AFspark] * Time [45]	0.57	−0.93 – 2.06	0.459	0.51	−0.08 – 1.09	0.089	0.88	−1.31 – 3.06	0.433
Condition [AFstill] * Time [60]	1.25	−0.52 – 3.02	0.167	0.11	−0.58 – 0.81	0.747	−1.25	−3.84 – 1.33	0.341
Condition [AFspark] * Time [60]	0.51	−1.26 – 2.28	0.569	0.34	−0.35 – 1.04	0.335	0.8	−1.79 – 3.38	0.546
Condition [AFstill] * Time [75]	1.22	−0.75 – 3.19	0.224	0.18	−0.61 – 0.96	0.653	−0.87	−3.75 – 2.01	0.552
Condition [AFspark] * Time [75]	0.09	−1.88 – 2.07	0.926	0.46	−0.32 – 1.25	0.248	1.81	−1.07 – 4.69	0.218
Condition [AFstill] * Time [90]	1.01	−1.12 – 3.14	0.353	0.19	−0.67 – 1.05	0.667	−0.94	−4.05 – 2.17	0.552
Condition [AFspark] * Time [90]	0.4	−1.73 – 2.53	0.713	0.4	−0.46 – 1.26	0.361	0.76	−2.36 – 3.87	0.634
Condition [AFstill] * Time [105]	1.46	−0.80 – 3.72	0.206	0.06	−0.86 – 0.98	0.894	−1.16	−4.45 – 2.14	0.49
Condition [AFspark] * Time [105]	0.03	−2.23 – 2.29	0.981	0.11	−0.81 – 1.03	0.816	1.29	−2.00 – 4.59	0.441
Condition [AFstill] * Time [120]	1.68	−0.68 – 4.05	0.162	−0.05	−1.02 – 0.92	0.92	−2.21	−5.65 – 1.24	0.209
Condition [AFspark] * Time [120]	0.52	−1.84 – 2.88	0.665	0.14	−0.83 – 1.11	0.771	0.13	−3.31 – 3.58	0.939
Condition [AFstill] * Time [135]	1.64	−0.81 – 4.09	0.189	0.02	−0.99 – 1.03	0.97	−2	−5.57 – 1.57	0.271
Condition [AFspark] * Time [135]	0.1	−2.35 – 2.55	0.937	0.19	−0.83 – 1.20	0.716	0.71	−2.86 – 4.28	0.697
Condition [AFstill] * Time [150]	1.44	−1.08 – 3.96	0.262	−0.03	−1.08 – 1.02	0.954	−1.18	−4.85 – 2.49	0.527
Condition [AFspark] * Time [150]	0	−2.52 – 2.51	0.997	0.16	−0.89 – 1.21	0.763	1.42	−2.25 – 5.09	0.447
